# COVID-19 vaccine hesitancy and associated factors according to sex: A population-based survey in Salvador, Brazil

**DOI:** 10.1371/journal.pone.0262649

**Published:** 2022-01-21

**Authors:** Nivison Nery, Juan P. Aguilar Ticona, Cristiane W. Cardoso, Ana Paula Pitanga Barbuda Prates, Helena Cristina Alves Vieira, Andrea Salvador de Almeida, Mirela Maisa da Silva Souza, Olivete Borba dos Reis, Maysa Pellizzaro, Moyra Machado Portilho, Renan Rosa da Anunciação, Renato Victoriano, Rosangela Oliveira dos Anjos, Hernán Dario Argibay, Douglas Oliveira Carmo Lima, Isadora Lima Mesquita, Wesley Mota Conceição, Perla Machado Santana, Elaine Carvalho Oliveira, Pamela Santos Nascimento Santana, Claudia Ida Brodskyn, Deborah Bittencourt Mothé Fraga, Manuela da Silva Solcà, Mitermayer Galvão Reis, Federico Costa, Guilherme S. Ribeiro

**Affiliations:** 1 Instituto Gonçalo Moniz, Fundação Oswaldo Cruz, Salvador, Brazil; 2 Instituto de Saúde Coletiva, Universidade Federal da Bahia, Salvador, Brazil; 3 Secretaria Municipal de Saúde de Salvador, Salvador, Brazil; 4 Escola de Medicina Veterinária e Zootecnia, Universidade Federal da Bahia, Salvador, Brazil; 5 Faculdade de Medicina, Universidade Federal da Bahia, Salvador, Brazil; 6 Yale School of Public Health, New Haven, CT, United States of America; Food and Drug Administration, UNITED STATES

## Abstract

Vaccination is a major strategy to prevent the coronavirus disease 2019 (COVID-19). However, information about factors associated with men and women intention to be vaccinated are scarce. To determine COVID-19 vaccine acceptance and identify factors associated vaccine hesitancy according to sex, we performed a cross-sectional population-based random survey in Salvador, Brazil between Nov/2020-Jan/2021. Participants were interviewed to obtain data on intention to receive and pay for a COVID-19 vaccine, as well as on demographics, comorbidities, influenza vaccination history, previous diagnosis of COVID-19, and exposures and perception of COVID-19 risk. Among 2,521 participants, 2,053 (81.4%) reported willingness to use a COVID-19 vaccine and 468 (18.6%) hesitated to take it. Among those intending to get vaccinated, 1,400 (68.2%) would pay for the vaccine if necessary. Sex-stratified multivariable analysis found that men who were working and who had comorbidities were less likely to hesitate about using the vaccine. Among women, higher educational level and high perception of COVID-19 risk were associated with less vaccine hesitancy. In both groups, reporting influenza vaccination in 2020 reduced the chance of COVID-19 vaccine hesitancy. COVID-19 vaccine campaigns targeting to reduce vaccine hesitancy are urgently needed. These campaigns should consider gender differences in order to be successful.

## Introduction

Given the continued transmission of Severe Acute Respiratory Syndrome Coronavirus-2 (SARS-CoV-2) worldwide and the lack of effective pharmacological measures against virus infection and disease, vaccination became a major strategy to prevent and control the coronavirus disease 2019 (COVID-19). However, despite the rapid development of COVID-19 vaccines in response to the pandemic, little information about intention to be vaccinated is available. Furthermore, some studies show an increasing trend towards vaccine hesitancy over time [1], which may be due to changes in perception of disease risk, spread of fake news, and misinformation [2].

Brazil is one of the countries most affected by the COVID-19 pandemic. In December 2020, a second wave of COVID-19 initiated in the north state of Amazonas, in association with the emergence of the Gamma (P1) variant of SARS CoV-2 [3]. From there, it spread across the country in the following months, attracting massive attention from researchers and the media due to the large number of cases and deaths in a population intensely exposed to SARS-CoV-2 during 2020 [4]. In this scenario, COVID-19 vaccination should be a priority, but, contrary to what was expected, by the end of 2020, the Brazilian government had not clearly declared its support for vaccination, generating uncertainty regarding vaccine use by the population [5–7]. Although COVID-19 vaccination had started in Brazil on January 17, 2021, political disputes around the use of the vaccine, lack of national campaigns supporting COVID-19 vaccination and informing about vaccine safety, and insufficient knowledge about factors that influence vaccine hesitancy may hamper vaccination coverage. Herein, we present population-based results on the intention to receive a COVID-19 vaccine and identify factors associated with vaccine hesitancy according to sex in Salvador, the fourth largest city in Brazil. These were secondary aims of a citywide survey that had as its primary goal to estimate the prevalence of antibodies against COVID-19, which is not presented in this article.

## Materials and methods

From November 16, 2020 to January 15, 2021, we conducted a door-to-door survey in Salvador, Brazil. Participants were household-based randomly selected using a cluster sampling method, as follow: in each of the 12 sanitary districts used to organize the city’s public health system, 50 postal codes (each representing one street) were randomly selected from a list of postal codes by health district. Then, five houses were systemically selected in each of the 50 selected streets within each sanitary district (the first house on the right of the street, followed by one house every three, until five houses were selected; if necessary, the same process was carried out on the left side of the street). In each of the selected houses, one of the residents fulfilling the inclusion criteria and who were available to participate in the survey at the time of the visit was drawn for inclusion in the study, aiming at a target sample of 3,000 participants.

Inclusion criteria for participation in the survey, which had as its primary goal to estimate the prevalence of antibodies against COVID-19, were age ≥5 years; living in the selected house, defined as sleeping ≥4 nights per week in the house; being at home at the time of the survey; and verbally consenting to participate prior to the drawing of one participant per house (after the draw, the selected participant signed the informed consent term). However, to accomplish the secondary aims of assessing the acceptance level of a COVID-19 vaccine, we restricted the analyzes to participants aged ≥18 years old and nonpregnant women; pregnant women were not included because at the time of the survey there were several concerns about vaccination during pregnancy. Participants were interviewed using a structured questionnaire (S1 and S2 Files) to obtain data on demographics, comorbidities (diabetes, hypertension, and cancer), influenza vaccination history, previous COVID-19 diagnoses, and exposures and perceptions of risk related to COVID-19. The intention to receive a COVID-19 vaccine was defined by a positive answer to the question: “If there was a safe and effective vaccine to prevent COVID-19, would you be interested in getting the vaccine?”. On the other hand, vaccine hesitancy regarding the use of a vaccine against COVID-19 was considered if the answer was “No”. In addition, we asked about the willingness to pay for the vaccine if necessary and the amount that participants would pay for it (five response options were provided in intervals of 50 Brazil real (BRL); ~8.80 US dollar (USD).

Data were summarized using descriptive statistics. To investigate differences between those who accepted and those who hesitated about COVID-19 vaccination, we used the chi-square test for dichotomous variables and the linear-by-linear test for ordinal variables. A p-value <0.05 was considered statistically significant. Then, because we found sex as a factor associated with vaccine hesitance, we repeated these analyses for male and female participants and used their results to choose variables (those with a p value < 0.20) to include in multivariable binomial logistic regression analyses, stratified by sex, to determine factors independently associated with vaccine hesitancy among men and among women. To measure associations, we used odds ratios (ORs) and 95% confidence intervals (CIs). The final model was built using a stepwise selection method, which used likelihood ratios as the criterion for adding significant variables to the model. We evaluated multicollinearity in final model by calculating the variance inflation factors (VIF), multicollinearity was defined if VIF > 10. The analysis was performed using R Statistical Software version 3.1.6.

The study was approved by the Ethics Committee of Instituto Gonçalo Moniz, Fundação Oswaldo Cruz (approval number 38468920.0.0000.0040). Written informed consent was obtained from all studied participants.

## Results

Among 2,537 nonpregnant participants aged ≥ 18 years, 2,521 (99.4%) had available data on intention to vaccinate against COVID-19. Of these, 2,053 (81.4%; 95% CI 79.9–82.9%) indicated willingness to accept a COVID-19 vaccine and 468 (18.6%; 95% CI 17.1–20.1%) hesitated in receiving the vaccine (S1 Table). Vaccine acceptance was higher among men (84.9%; 95% CI 82.1–87.0%) compared to women (79.7%; 95% CI 77.7–81.6%). Among the participants who accepted to be vaccinated, 1,400 (68.2%) reported an intention to pay if necessary. Of these, the majority (778; 55.6%) would be willing to pay up to 50 BRL (8.8 USD) and only 160 (11.4%) reported that they would pay more than 200 BRL (35.4 USD) (S1 Table). Willingness to pay and the amount to pay, if necessary, did not differ by sex.

Bivariate sex-stratified analysis showed some differences between men and women (Table 1). Among men, increasing age and reporting comorbidities reduced the likelihood of COVID-19 vaccine hesitancy. On the other hand, among women, self-reported white ethnicity, higher educational level, report of previous symptoms compatible with COVID-19, report of previous testing for COVID-19, and having a greater perception of risk of contracting COVID-19 reduced the likelihood of hesitancy about vaccination. History of influenza vaccination in 2020 was the only variable that positively influenced the intention to vaccinate both among men and women.

**Table 1:** Participants’ characteristics and intention to receive a COVID-19 vaccine among men and among women, Salvador, Brazil.

Characteristics	Intention to receive a COVID-19 vaccine among men			p value	Intention to receive a COVID-19 vaccine among women			p value
	Total	Yes	No		Total	Yes	No	
	N = 834	n = 708	n = 126		N = 1,687	n = 1,345	n = 342	
	n (column %)	n (row %)			n (column %)	n (row %)		
**Demographics**								
Age	Response = 833 ^1^			**0.006** ^2^	Response = 1,687			0.336 ^2^
< 40	252 (30.3%)	200 (79.4%)	52 (20.6%)		522 (30.9%)	407 (78.0%)	115 (22.0%)	
40–65	435 (52.2%)	376 (86.4%)	59 (13.6%)		853 (50.6%)	692 (81.1%)	161 (18.9%)	
> 65	146 (17.5%)	132 (90.4%)	14 (9.6%)		312 (18.5%)	246 (78.8%)	66 (21.2%)	
Ethnicity	Response = 833 ^1^			0.964	Response = 1,686^1^			**0.037**
White	70 (8.4%)	60 (85.7%)	10 (14.3%)		134 (7.9%)	118 (88.1%)	16 (11.9%)	
Black	329 (39.5%)	277 (84.2%)	52 (15.8%)		691 (41.0%)	556 (80.5%)	135 (19.5%)	
Brown	410 (49.2%)	350 (85.4%)	60 (14.6%)		821 (48.7%)	642 (78.2%)	179 (21.8%)	
Others	24 (2.9%)	20 (83.3%)	4 (16.7%)		40 (2.4%)	29 (72.5%)	11 (27.5%)	
Years of formal education	Response = 834			0.589	Response = 1,686 ^1^			**0.003**
0 to 9	247 (29.6%)	210 (85.0%)	37 (15.0%)		520 (30.8%)	404 (77.7%)	116 (22.3%)	
10 to 12	436 (52.3%)	366 (83.9%)	70 (16.1%)		839 (49.8%)	658 (78.4%)	181 (21.6%)	
> 12	151 (18.1%)	132 (87.4%)	19 (12.6%)		327 (19.4%)	283 (86.5%)	44 (13.5%)	
Married or stable union	Response = 833 ^1^			0.094	Response = 1,686^1^			0.769
Yes	391 (46.9%)	341 (87.2%)	50 (12.8%)		642 (38.1%)	515 (80.2%)	127 (19.8%)	
No	442 (53.1%)	366 (82.8%)	76 (17.2%)		1,044 (61.9%)	830 (79.5%)	214 (20.5%)	
Currently working	Response = 834			0.125	1,686			0.389
Yes	400 (48.0%)	348 (87.0%)	52 (13.0%)		583 (34.6%)	472 (81.0%)	111 (19.0%)	
No	434 (52.0%)	360 (82.9%)	74 (17.1%)		1,103 (65.4%)	872 (79.1%)	231 (20.9%)	
Health professional	Response = 833 ^1^			>0.999	Response = 1,682 ^1^			0.106
Yes	25 (3.0%)	21 (84.0%)	4 (16.0%)		74 (4.4%)	65 (87.8%)	9 (12.2%)	
No	808 (97.0%)	686 (84.9%)	122 (15.1%)		1,608 (95.6%)	1,277 (79.4%)	331 (20.6%)	
N° of household residents	Response = 782			0.405 ^2^	1,596			0.996 ^2^
01–02	339 (43.4%)	292 (86.1%)	47 (13.9%)		622 (39.0%)	497 (79.9%)	125 (20.1%)	
03–04	345 (44.1%)	292 (84.6%)	53 (15.4%)		753 (47.2%)	603 (80.1%)	150 (19.9%)	
> 4	98 (12.5%)	79 (80.6%)	19 (19.4%)		221 (13.8%)	177 (80.1%)	44 (19.9%)	
**COVID-19 diagnoses and experiences**								
Experienced COVID-19 symptoms	Response = 834			0.772	Response = 1,687			**0.033**
Yes	244 (29.3%)	209 (85.7%)	35 (14.3%)		644 (38.2%)	531 (82.5%)	113 (17.5%)	
No	590 (70.7%)	499 (84.6%)	91 (15.4%)		1,043 (61.8%)	814 (78.0%)	229 (22.0%)	
Believe that had COVID-19	831			0.373	Response = 1,682 ^1^			0.271
Yes	138 (16.6%)	121 (87.7%)	17 (12.3%)		370 (22.0%)	303 (81.9%)	67 (18.1%)	
No	693 (83.4%)	584 (84.3%)	109 (15.7%)		1,312 (78.0%)	1,038 (79.1%)	274 (20.9%)	
Received a medical diagnosis of COVID-19	Response = 834			0.676	Response = 1,685 ^1^			0.228
Yes	31 (3.7%)	25 (80.6%)	6 (19.4%)		66 (3.9%)	57 (86.4%)	9 (13.6%)	
No	803 (96.3%)	683 (85.1%)	120 (14.9%)		1,619 (96.1%)	1,287 (79.5%)	332 (20.5%)	
Previously tested to COVID-19	Response = 834			0.781	Response = 1,686^1^			**0.014**
Yes	109 (13.1%)	94 (86.2%)	15 (13.8%)		172 (10.2%)	150 (87.2%)	22 (12.8%)	
No	725 (86.9%)	614 (84.7%)	111 (15.3%)		1,514 (89.8%)	1,195 (78.9%)	319 (21.1%)	
Hospitalization	Response = 834			0.749	Response = 1,687			>0.999
Yes	5 (0.6%)	5 (100.0%)	0 (0.0%)		10 (0.6%)	8 (80.0%)	2 (20.0%)	
No	829 (99.4%)	703 (84.8%)	126 (15.2%)		1,677 (99.4%)	1,337 (79.7%)	340 (20.3%)	
Admission to an ICU	Response = 834			0.749	1,687			>0.999
Yes	5 (0.6%)	5 (100.0%)	0 (0.0%)		12 (0.7%)	10 (83.3%)	2 (16.7%)	
No	829 (99.4%)	703 (84.8%)	126 (15.2%)		1,675 (99.3%)	1,335 (79.7%)	340 (20.3%)	
**COVID-19 exposures at household**								
Household member suspected of COVID-19	Response = 782 ^1^			0.355	Response = 1,599 ^1^			0.063
Yes	109 (13.9%)	96 (88.1%)	13 (11.9%)		294 (18.4%)	247 (84.0%)	47 (16.0%)	
No	673 (86.1%)	566 (84.1%)	107 (15.9%)		1,305 (81.6%)	1,031 (79.0%)	274 (21.0%)	
Hospitalization of a household member suspected of COVID-19	Response = 832 ^1^			>0.999	Response = 1,679 ^1^			0.378
Yes	8 (1.0%)	7 (87.5%)	1 (12.5%)		26 (1.5%)	23 (88.5%)	3 (11.5%)	
No	824 (99.0%)	699 (84.8%)	125 (15.2%)		1,653 (98.5%)	1,314 (79.5%)	339 (20.5%)	
Death of a household member suspected of COVID-19	Response = 781 ^1^			>0.999	Response = 1,591			>0.999
Yes	2 (0.3%)	2 (100.0%)	0 (0.0%)		7 (0.4%)	6 (85.7%)	1 (14.3%)	
No	779 (99.7%)	659 (84.6%)	120 (15.4%)		1,584 (99.6%)	1,264 (79.8%)	320 (20.2%)	
Type of household	782			0.670	Response = 1,596 ^1^			0.514
House	719 (91.9%)	607 (84.4%)	112 (15.6%)		1,454 (91.1%)	1,159 (79.7%)	295 (20.3%)	
Apartment	63 (8.1%)	55 (87.3%)	8 (12.7%)		142 (8.9%)	117 (82.4%)	25 (17.6%)	
**Comorbidities**	834			**0.004**	Response = 1,687			0,279
Yes	274 (32.9%)	247 (90.1%)	27 (9.9%)		692 (41.0%)	561 (81.1%)	131 (18.9%)	
No	560 (67.1%)	461 (82.3%)	99 (17.7%)		995 (59.0%)	784 (78.8%)	211 (21.2%)	
Received influenza vaccine in 2020	Response = 810 ^1^			**0.006**	Response = 1,650 ^1^			**<0.001**
Yes	421 (52.0%)	374 (88.8%)	47 (11.2%)		946 (57.3%)	791 (83.6%)	155 (16.4%)	
No	389 (48.0%)	318 (81.7%)	71 (18.3%)		704 (42.7%)	527 (74.9%)	177 (25.1%)	
**COVID-19 risk perception**								
How likely are you to get the the COVID-19?	Response = 834			0.576	Response = 1,687			**0.043**
Not probable	113 (13.5%)	96 (85.0%)	17 (15.0%)		261 (15.5%)	195 (74.7%)	66 (25.3%)	
Slightly or Moderately probable	568 (68.1%)	478 (84.2%)	90 (15.8%)		1,178 (69.8%)	943 (80.1%)	235 (19.9%)	
Very probable	153 (18.3%)	134 (87.6%)	19 (12.4%)		248 (14.7%)	207 (83.5%)	41 (16.5%)	
How severe can COVID-19 be?	Response = 832 ^1^			0.062	Response = 1,684 ^1^			0.108
Not serious	158 (19.0%)	135 (85.4%)	23 (14.6%)		323 (19.2%)	245 (75.9%)	78 (24.1%)	
A little or Moderate serious	570 (68.5%)	475 (83.3%)	95 (16.7%)		1,102 (65.4%)	883 (80.1%)	219 (19.9%)	
Very serious	104 (12.5%)	96 (92.3%)	8 (7.7%)		259 (15.4%)	214 (82.6%)	45 (17.4%)	

NOTE: Bold values indicate statistically significant associations (p < 0.05).

^1^ Some variables have a lower number of responses due to unavailability of data.

^2^ Linear-by-linear association test.

**Fig 1:**
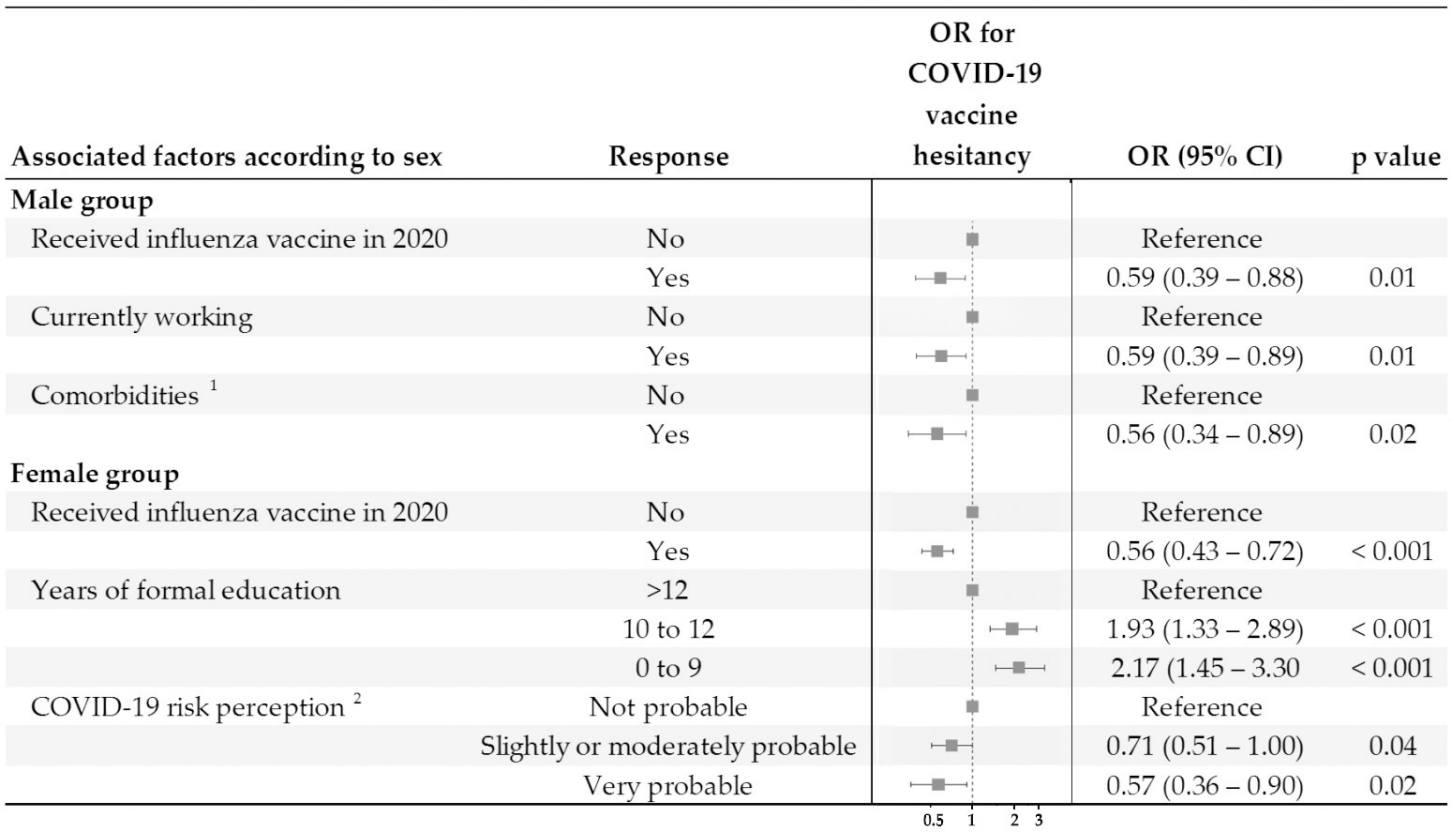
Sex-stratified multivariable analysis for identification of factors associated with COVID-19 vaccine hesitancy among men and among women, Salvador, Brazil, 16 November 2020–15 January 2021. ^1^ Comorbidities included diabetes, hypertension, and cancer. ^2^ Risk perception evaluated using the question: How likely are you to get COVID-19? OR: Odds Ratio.

After running stepwise multivariable regression models for men and women separately, the final models showed that a history of influenza vaccination in 2020 reduced the chance of COVID-19 vaccine hesitancy similarly for men (OR = 0.59; 95% CI 0.39–0.88) and women (OR = 0.56; 95% CI 0.43–0.72) (Fig 1). However, other factors that were independently associated with COVID-19 vaccine hesitancy differed by sex (Fig 1). Among men, those who were working (OR = 0.59; 95% CI 0.39–0.89), and who reported comorbidities (OR = 0.56; 95% CI 0.34–0.89) were less likely to have an attitude of hesitancy towards COVID-19 vaccine. Among women, those with complete or incomplete high school (10–12 years of study) (OR = 1.93; 95% CI 1.33–2.89) and those with complete or incomplete elementary or middle school (0–9 years of study) (OR = 2.17; 95% CI 1.45–3.30) had an increased chance of hesitancy in relation to vaccination, compared to those with at least some level of higher education (>12 years of study). In addition, women who thought they had a moderate (OR = 0.71; 95% CI 0.51–1.00) or high risk (OR = 0.57; 95% CI 0.36–0.90) of having COVID-19 were less likely to hesitate about COVID-19 vaccination.

## Discussion

Our citywide survey, conducted prior to the initiation of the COVID-19 vaccination campaigns in Brazil, found a relatively high rate of intention to receive a COVID-19 vaccine (81.4%) in a large Brazilian urban center. It also identified that men and women had different factors associated with hesitancy regarding receiving a COVID-19 vaccine.

It has been shown that the context of SARS-CoV-2 transmission can determine the population’s intention to be vaccinated, according to the perception of risk [1, 8]. Our study was initiated five months after the first COVID-19 peak in Salvador, in June 2020 [9, 10], and completed before the second wave peak, in February 2021 [11]. Thus, our survey was carried out in a period of flexibilization of measures of social distancing, when the highest levels of population mobility were registered [12] and the population’s perception of risk was probably lower.

Likewise, an online survey conducted in Brazil around the peak of the first wave of COVID-19, in June 2020, found that 85% (95% CI 83–88%) of the general population reported a willingness to receive a COVID-19 vaccine [8]. The relatively high rate of intention to vaccinate observed in both studies is surprising, given the very different epidemiological contexts, the political crisis surrounding the Brazilian government’s management of the pandemics [5–7] and all the population’s misinformation about COVID-19 treatment and vaccination [13].

In our study, female sex was associated with hesitancy regarding the intention to use a COVID-19 vaccine. However, the role of gender in COVID-19 vaccine hesitancy seems to vary, depending on the social and cultural background of the studied population. Previous studies have highlighted that woman tended to perceive themselves at risk for COVID-19 more often than men and that they were also more likely to accept a COVID 19 vaccine [8, 14]. In contrast, a large online survey conducted in the United Kingdom in 2020 found that female gender was associated with COVID-19 vaccine hesitancy [15]. In addition, a meta-analysis evaluating gender differences related to preventive practices during respiratory epidemics, including Middle East respiratory syndrome (MERS) and SARS, found that women have a greater acceptance of non-pharmacological preventive practices, such as hand washing and crowd avoidance, while men had a slightly greater acceptance of pharmacological preventive practices, including the willingness to be vaccinated [14, 16]. Another meta-analysis found that among both the general population and health professionals, men were more likely to be vaccinated and also had a higher intention to be vaccinated against influenza virus than women [17]. Moreover, women expressed more concerns about the efficacy and safety of the influenza vaccine than men [17].

As expected, we found that both men and women who got the influenza vaccine during 2020 had lower rates of COVID-19 vaccine hesitancy, suggesting that they had a vaccine-prone behavior. In addition, we also found gender-specific factors associated with vaccine hesitancy. Men who were working were less likely to hesitate to get vaccinated, probably because they fear losing their job or having to stop if they get sick, a fear that may be reinforced by the economic crisis and the rising unemployment rate associated with the COVID-19 pandemic. It was also shown that 48.1% of people who would accept a COVID-19 vaccine considered the employer’s recommendation in their decision [8]. Men who reported comorbidities were also less likely to be hesitant about using a COVID-19 vaccine, which is understandable given the continuous announcement that comorbidities significantly increase hospitalization and mortality rates due to COVID-19 [18–20], and that those with comorbidities should be prioritized for COVID-19 vaccination. It is also possible that these two factors, having a work and having comorbidities, operated together to reduce the likelihood of vaccine hesitancy, because people with comorbidities and working may be the ones who most need to continue healthy to keep their jobs. Similar to what other studies have shown, among women, lower educational level increased the likelihood of vaccine hesitancy [8], while a higher perceived risk of having COVID-19 [14, 21] reduced the chance of vaccine hesitancy.

The finding that both presence of comorbidities among men and having a higher perceived risk of having COVID-19 among women reduced the likelihood of COVID-19 vaccine hesitancy may suggest that personal concerns about developing severe forms of the disease are important motivators for vaccine acceptance. This is consistent with results from a large, well-designed, randomized clinical trial conducted in the United Kingdom, which assessed whether brief statements about vaccination against COVID-19 would reduce vaccine hesitancy [22]. Interestingly, the study found that among those strongly hesitant about COVID-19 vaccination, providing statements addressing potential personal benefits of vaccination had a greater impact on reducing hesitancy than statements containing information about the potential collective benefits of vaccination [22, 23]. Understanding the type of message content that can change the intention of being vaccinated is essential for public health agents engaged in designing campaigns to encourage COVID-19 vaccination.

Previous studies found that hesitancy about vaccination against COVID-19 and influenza vaccination was more common among younger individuals [1, 8, 24]. We observed the same association for males in the bivariate analysis; however, in the multivariable analysis, age was not important for the model and was excluded during the process of variable selection. Nevertheless, as comorbidities are more frequent among the elderly and because influenza vaccination campaigns in Brazil have people older than 60 years of age and with previous comorbidities as target groups [25, 26], it was possible that the found associations between vaccine hesitancy and both a history of comorbidities and prior influenza vaccination were somehow confounded by another age-related factor. To rule out this possibility, we forced age-adjustment in the final models built for men and for women, and all previously detected associations remained statistically significant, except for comorbidities among men, which became marginally associated (P = 0.064). These findings support that prior use of an influenza vaccine and having comorbidities are truly correlated with reduced hesitancy to the COVID-19 vaccine, influencing people’s motivation and attitudes towards vaccination.

The Brazilian Unified Health System (Sistema Único de Saúde - SUS) has several success stories in implementing health programs [27]. Among them, the National Immunization Program (Programa Nacional de Imunizações—PNI), which offers nearly 20 universally free vaccines for children, adolescents, adults, and the elderlies, is a milestone [28]. Interestingly, our study found a high intention to pay for a COVID-19 vaccine if needed. This finding raises concerns regarding the potential negative effects of allowing the private sector to distribute COVID-19 vaccines in the country, especially considering the slow vaccination rates, the difficulties in acquiring and producing COVID-19 vaccines in Brazil, and, as shown in this work, the huge population demand for a vaccine. Given the enormous social disparity that exists in Brazil and the universally free nature of the Brazilian PNI, authorizing the unrestricted trade in vaccines against COVID-19 may deepen the major health inequity problems already present in the country and hinder access to the vaccine for priority groups that couldn’t pay for the vaccine.

Our study has some limitations. As a cross-sectional study, it was not possible to assess any changes in vaccine hesitancy rates that may have occurred due to the increase in COVID-19 cases and deaths during the second epidemic wave or due to the start of COVID-19 vaccination campaigns in Brazil [3, 5]. Furthermore, although the survey had been carried out in a cluster-based random sample, the selection of participants within the selected households was carried out among those who were at home during the research team’s visit, during working hours on weekdays. Thus, the final sample was likely less representative of the working population, and possibly biased the estimated rate of intention to receive a COVID-19 vaccine by reducing it, as we found that having a work was associated with a lower likelihood of vaccine hesitancy. The selection of participants in the selected households can also explain the higher frequency of women in our sample. To minimize possible misinterpretations in the investigation of factors associated with vaccine hesitancy, we opted to perform sex-stratified analyses and report the multivariable analyses results by sex. Another potential limitation is related to the question used to assess acceptance of vaccination, as it refers to a context in which vaccination proved to be safe and efficacious. However, at the time of the survey, there were concerns about the efficacy and safety of COVID-19 vaccines [2, 6, 29], and therefore our question may have induced a positive response to vaccine acceptance. We adopted this question because it is similar to questions previously used by other studies, some of which with Brazilian participants [1, 8, 29]. Further studies should assess the acceptance of COVID-19 vaccines in a context where they are being widely used.

## Conclusions

We found a relatively low rate of COVID-19 vaccine hesitancy following the first COVID-19 epidemic peak and before the second wave peak in a large Brazilian city. Gender was a factor associated with vaccine hesitance and most other associated factors differed between men and women. Thus, based on this survey performed in Salvador, Brazil, differences between men and women in vaccine hesitancy rate should be considered when defining health policies and designing campaigns to promote COVID-19 vaccination and reduce vaccine refusal rates. Additional studies are warranted to evaluate and monitor specific concerns about COVID-19 vaccination and antivaccine attitudes. In addition, longitudinal studies should be carried out because the perception of COVID-19 risk, a factor that we found to be associated with vaccine hesitancy among women, can change over time, according to the COVID-19 epidemiological context, which can affect the intention to be vaccinated.

## Supporting information

S1 File Survey instrument in English. (DOCX)

S2 File Survey instrument in Portuguese. (DOCX)

S1 Table Intention to receive a COVID-19 vaccine and willingness to pay if needed, stratified by sex, Salvador, Brazil. (DOCX)

S1 Database Excel database containing the data used in the tables and figure. (XLS)
