# Supplementary material for: COVID-19 vaccine hesitancy and associated factors according to sex: A population-based survey in Salvador, Brazil
Source: PLoS One. 2022 Jan 21;17(1):e0262649. doi: 10.1371/journal.pone.0262649 (PMC8782400; doi:10.1371/journal.pone.0262649)

Individual Questionnaire

Study identification number:

(Adhesive label)

**INCLUSION IN THE STUDY**

Interview date:

Address:

Type of residence:


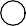
 House
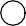
 Apartment

Do you confirm your interest in participating in the survey?


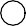
 Yes
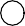
 No

**PARTICIPANT IDENTIFICATION**

Could you confirm your full name for me?

What is your date of birth?

How old are you?

Participant's gender:


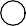
 Male
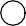
 Female

What is your current marital status?


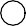
 Single
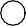
 Married
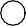
 stable union > 6 months
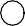
 Separated or divorced
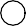
 Widower

What color skin do you identify with?

White
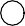
 Black
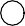
 Brown (mulatto, brown, mixed...)
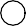
 Yellow
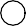
 Indigenous
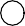
 don't know another


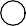

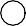


If another, which one?

Were you studying before the quarantine started?


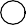
 Yes
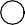
 No

What was the highest grade or level of school that you completed?

- Never studied
- Literacy (1 year)
- 1st Grade (2 year)
- 2nd Grade (3 years)
- 3rd Grade (4 years)
- 4th Grade (5 years)
- 5th Grade (6 years)
- 6th Grade (7 years)
- 7th Grade (8 years)
- 8th Grade (9 years)
- 1 year of High School
- 2 year of high school
- 3 year of high school
- Higher education incomplete
- Higher education complete

Are you currently working or developing any paid activity (formal or informal)?


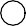
 Yes
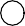
 No

If yes, what activity/occupation?

How many people live in the house, including you?

(NOTE: only count those who, in the last 6 months, have slept at least 3 days a week at home)

**COVID-19 AND HEALTH CONDITIONS**

Has a doctor ever told you that you had or have any of the following diseases? (You can select more than one)

hipertension diabetes cancer

Coronary (disease (history of angina, heart attack, stent placement, myocardial revascularization)

Heart failure

Arrhythmia

Stroke (stroke, stroke)

Chronic obstructive Chronic obstructive pulmonary disease (COPD)

Asthma

Obesity

Dyslipidemia

Depression

Dengue Zika

Chikungunya

Any other disease

None

Did you get vaccinated against flu (influenza) in 2020?
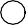
 Yes
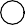
 No
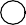
 Do not remember

Do you think you had COVID-19?


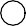
 Yes
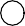
 No

Have you been told by a doctor that you have had Covid-19?


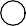
 Yes
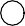
 No

Have you had any lab tests to find out if you have or had Covid-19?


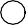
 Yes
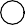
 No

Since when the Covid-19 epidemic started in Salvador, in March, have you had any of the following symptoms?

Cough

Rhinorrhea

Sore throat

Shortness of breath fever

Chills

Headache

Body Pain

Joint Pain Diarrhea

Nausea Vomiting Irritability

Tiredness/weakness Redness in the eyes

Red spots on the skin

Abdominal pain

Chest pain

Loss of taste (no longer tasting food)

Loss of smell (no longer smelling)

Nasal congestion (stuffy nose)

None of the symptoms above

Have you seen a doctor due to these symptoms?


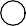
 Yes
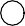
 No

If you have sought medical care, have you had to stay in hospital for more than 24 hours?
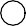
 Yes
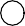
 No

If you were in the hospital for more than 24 hours, did you need to be treated in an intensive care unit or semi-intensive care unit?

Yes, in ICU


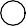

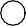

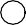

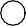


Yes, in semi-ICU

Yes, in ICU and semi-ICU No

**HABITS/BEHAVIORS/PRACTICES DURING THE PANDEMIC AND RISK EXPOSURES**

Had any of the inhabitants of your household (not counting you) been suspected of COVID-19?


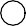
 Yes
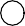
 No

Have any of them been hospitalized, how many?

Did any of them die, how many?

Do you work in the health sector?

(Health workers are considered to be health, sanitation and administrative professionals)


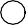
 Yes
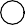
 No

4.4. Risk perception

In your opinion, what is the possibility of you contracting COVID-19 in the future?


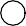
 not possible
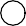
 low possibility
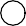
 moderate possibility
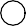
 high possibility

In your opinion, how serious do you think you would be if you had COVID19?


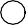
 Nothing severe
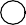
 not serious
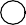
 moderately severe
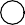
 Very serious

If there was a safe and effective vaccine to prevent COVID, would you be interested in using it?


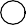
 Yes
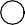
 No

If you had to pay for the vaccine, would you be willing to buy it at an affordable price?


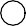
 Yes
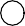
 No

How much would you be willing to pay for a dose of the vaccine?

Up to 50 reais
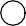
 Between 51 and 100 reais
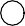
 Between 101 and 150 reais Between 151 and 200 reais
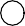
 More than 200 reais


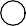

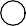

Supplement: S1 File — (DOCX) [file pone.0262649.s001.docx]
