# Supplementary material for: COVID-19 vaccine hesitancy and associated factors according to sex: A population-based survey in Salvador, Brazil
Source: PLoS One. 2022 Jan 21;17(1):e0262649. doi: 10.1371/journal.pone.0262649 (PMC8782400; doi:10.1371/journal.pone.0262649)

Questionario Individual

Numero de identificacao no estudo:

(Etiqueta)

**INCLUSAO NO ESTUDO**

Data da Entrevista:

Endereco:

Tipo de residencia:


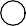
 Casa
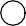
 Apartamento

O(A) senhor(a) me confirma o interesse em participar da pesquisa?
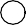
 Sim
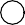
 Nao

**IDENTIFICACAO DO PARTICIPANTE**

O(A) senhor(a) poderia me confirmar o seu nome completo?

O(A) senhor(a) poderia me informar a sua data de nascimento?

Quantos anos o(a) Senhor(a) tem?

Registrar o sexo do participante:
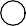
 Masculino
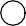
 Feminino

Qual seu estado civil atual?


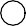
 Solteiro
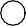
 Casado legalmente
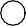
 Uniao estavel ha > 6 meses
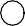
 Separado ou divorciado
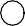
 Viuvo

O(A) senhor(a) se considera de que cor/raa?

Branco
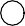
 Preto
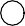
 Pardo (mulato, mestico, moreno...)
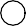
 Amarelo
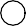
 Indigena
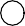
 Nao sabe Outra


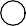

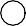


Se outra, qual?

O(A) senhor(a) estava estudando antes da quarentena ser iniciada?
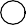
 Sim
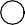
 Nao

Qual foi a ltima srie escolar que o(a) senhor(a) completou?

Nunca estudou Alfabetizacao (1 ano) 1a Serie (2 ano) 2a Serie (3 ano) 3a Serie (4 ano) 4a Serie (5 ano) 5a Serie (6 ano) 6a Serie (7 ano) 7a Serie (8 ano) 8a Serie (9 ano) 1 ano do Ensino Medio
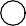
 2 ano do Ensino Medio
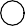
 3 ano do Ensino Medio Nivel Superior


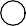

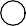

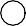

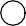

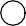

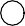

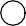

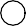

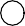

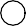

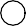

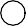


incompleto
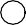
 Nivel Superior completo

Atualmente, o(a) Sr(a) est trabalhando ou desenvolve alguma atividade (formal ou informal) remunerada?
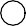
 Sim
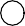
 Nao

Se sim, qual atividade/ocupacao?

Quantas pessoas moram na casa, incluindo o(a) Sr(a)?

(OBS: contabilizar apenas aqueles que nos ultimos 6 meses tem dormido pelo menos 3 dias por semana no domicilio)

**COVID-19 E CONDICOES DE SAUDE**

Alguma vez algum medico lhe disse que o(a) senhor(a) teve ou tem alguma das seguintes doencas? (Pode selecionar mais que uma)

Pressao alta Diabetes Cancer

Doenca arterial coronariana (historico de angina, infarto, colocacao de stent, revascularizacao miocardica) Insuficiencia cardiaca

Arritmia cardiaca

Acidente vascular cerebral (AVC, derrame) Doenca pulmonar obstrutiva cronica (DPOC) ASMA

Obesidade

Dislipidemia (colesterol ou triglicerides alto) Depressao

Dengue Zika

Chikungunya

Alguma outra doença

Nenhuma

O(A) Sr(a) se vacinou contra gripe (influenza) em 2020?
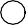
 Sim
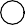
 Nao
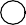
 Nao lembra

O(A) Sr(a) acha que teve Covid-19?
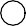
 Sim
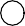
 Nao

Algum medico informou ao Sr(a) que voce teve Covid-19?
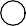
 Sim
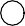
 Nao

O(A) Sr(a) realizou algum exame laboratorial para saber se estava ou teve Covid-19?
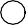
 Sim
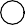
 Nao

Desde quando a epidemia de Covid-19 se iniciou em Salvador, em marco, o (a) Sr(a) apresentou algum dos seguintes sintomas?

Tosse Coriza

Dor de garganta Falta de ar Febre

Calafrio

Dor de cabeca Dores no corpo Dores nas juntas Diarreia

Nausea Vomito Irritabilidade

Cansaco/fraqueza Vermelhidao nos olhos Manchas vermelhas na pele Dor abdominal

Chiado no peito

Perda do paladar (deixou de senti o sabor dos alimentos) Perda do olfato (deixou de sentir cheiros)

Congestao nasal (nariz entupido) Nenhum dos sintomas acima

Por causa destes sintomas, o(a) sr(a) buscou atendimento medico?
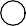
 Sim
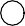
 Nao

Se buscou atendimento medico, precisou ficar hospitalizado por mais de 24h?
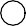
 Sim
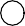
 Nao

Se foi hospitalizado por mais de 24h, precisou de tratamento em UTI ou semi-UTI?

Sim, em UTI


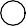

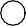

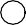

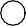


Sim, em semi UTI

Sim, em UTI e semi UTI Nao

**HABITOS/COMPORTAMENTOS/ PRATICAS DURANTE A PANDEMIA E EXPOSICOES DE**

**RISCO**

Algum(a) morador(a) de sua casa (sem contar o(a) senhor(a)) teve suspeita de COVID-19?
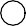
 Sim
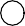
 Nao

Algum deles foi hospitalizado, quantos?

Algum deles faleceu, quantos?

O(A) senhor(a) trabalha na area de sade?

(Sao consideradores trabalhadores da saude os profissionais de saude, da higienizacao e admistrativo)
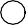
 Sim
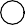
 Nao

4.4. Percepcao de risco

Em sua opiniao, qual a possibilidade do sr(a) contrair a COVID-19 no futuro?


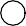
 Nenhuma possibilidade
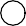
 Baixa possibilidade
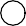
 Moderada possibilidade
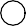
 Alta possibilidade

Em sua opiniao, quao grave o sr(a) acha que ficaria se tivesse o COVID19?


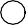
 Nada grave
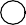
 Pouco grave
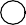
 Moderadamente grave
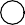
 Muito grave

Se houvesse uma vacina segura e efetiva para prevenir a COVID, voce teria interesse em usar?
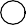
 Sim
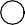
 Nao

Se voce tivesse que pagar pela vacina, voce estaria disposto a pagar caso ela custasse um valor acessivel para voce?


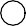
 Sim
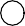
 Nao

Ate quanto estaria disposto a pagar por uma dose da vacina?

Ate 50 reais
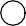
 Entre 51 e 100 reais
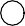
 Entre 101 e 150 reais
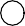
 Entre 151 e 200 reais Mais de 200 reais


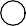

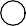

Supplement: S2 File — (DOCX) [file pone.0262649.s002.docx]
