# Supplementary material for: COVID-19 vaccine hesitancy and associated factors according to sex: A population-based survey in Salvador, Brazil
Source: PLoS One. 2022 Jan 21;17(1):e0262649. doi: 10.1371/journal.pone.0262649 (PMC8782400; doi:10.1371/journal.pone.0262649)
Supplement: S1 Table — (DOCX) [file pone.0262649.s003.docx]

Table S1. Intention to receive a COVID-19 vaccine and willingness to pay if needed, stratified by sex, Salvador, Brazil.

| **Characteristic** | **Total  N = 2,521** | **Men  n = 834** | **Women n = 1,687** |
| --- | --- | --- | --- |
|  | **n (%)** | | |
| Intention to receive a COVID-19 vaccine | Response = 2,521 |  |  |
| Yes | 2,053 (81.4) ^1^ | 708 (84.9) ^2^ | 1,345 (79.7) ^3^ |
| No | 468 (18.6) ^4^ | 126 (15.1) ^5^ | 342 (20.3) ^6^ |
| Willingness to pay if needed ^7^ | Response = 2,051 |  |  |
| Yes | 1,400 (68.3) | 482 (68.2) | 918 (68.3) |
| No | 651 (31.7) | 225 (31.8) | 426 (31.7) |
| Value that would pay if needed ^8^ | Response = 1,400 |  |  |
| Up to R$50 | 778 (55.6) | 267 (55.4) | 511 (55.7) |
| Between R$51 and R$100 | 279 (19.9) | 93 (19.3) | 186 (20.3) |
| Between R$101 and R$150 | 71 (5.1) | 27 (5.6) | 44 (4.8) |
| Between R$151 and R$200 | 112 (8.0) | 30 (6.2) | 82 (8.9) |
| More than R$200 | 160 (11.4) | 65 (13.5) | 95 (10.3) |

^1^ 95% CI = 79.9 – 82.9%.

^2^ 95% CI = 82.3 – 87.2%.

^3^ 95% CI = 77.7 – 81.6 %.

^4^ 95% CI = 17.1 – 20.1%.

^5^ 95% CI = 12.8 – 17.7 %.

^6^ 95% CI = 18.4 – 22.3%.

^7^ For 2,051 participants who reported intention to receive a COVID-19 vaccine. Data was missing for 2 participants.

^8^ For 1,400 participants who reported willingness to pay for a vaccine.
